# Identification of DAP3 as candidate prognosis marker and potential therapeutic target for hepatocellular carcinoma

**DOI:** 10.3389/fimmu.2025.1528853

**Published:** 2025-02-20

**Authors:** Liu-Xia Yuan, Zhi-Qiang Yue, Qin-Rong Ma, Peng Zhang, Feng Xiao, Lin Chen

**Affiliations:** ^1^ Institute of Liver Diseases, Nantong Third People’s Hospital, Affiliated Nantong Hospital 3 of Nantong University, Nantong, Jiangsu, China; ^2^ Department of Hepatobiliary Surgery, Nantong Third People’s Hospital, Affiliated Nantong Hospital 3 of Nantong University, Nantong, Jiangsu, China; ^3^ Department of Pathology, Nantong Third People’s Hospital, Affiliated Nantong Hospital 3 of Nantong University, Nantong, Jiangsu, China

**Keywords:** hepatocellular carcinoma, DAP3, prognostic model, bioinformatics, biomarker

## Abstract

**Background:**

Among malignant tumors, hepatocellular carcinoma (HCC) is both prevalent and highly lethal. Most patients with advanced-stage liver cancer have a poor prognosis. Death-associated protein 3 (DAP3) is reportedly related to tumors and may hold great promise for the future.

**Methods:**

DAP3 transcriptome data along with related clinical information were obtained from The Cancer Genome Atlas (TCGA), GEO, and ICGC databases. We assessed its prognostic value, clinical relevance, associated pathways, immune infiltration, gene mutations, and sensitivity to chemotherapeutics. A prognostic risk model was subsequently developed and evaluated using receiver operating characteristic (ROC) curves and Kaplan-Meier (KM) plots. Additionally, a nomogram was created and validated through calibration and decision curve analysis (DCA). Furthermore, quantitative real-time PCR (qRT-PCR), Western blot, and immunohistochemical (IHC) staining were performed to examine the expression of DAP3 in HCC. Finally, gene knockdown and overexpression experiments, along with cell counting kit-8 (CCK-8) assays, colony formation assays, and tests for cell apoptosis, migration, and invasion, were conducted to investigate the role of DAP3 in HCC.

**Results:**

The study discovered that DAP3 expression was linked to HCC subtypes, and its high expression was linked to a poor prognosis. There were significant differences in immune infiltration level, mutation level, prognostic value and chemotherapeutic efficacy. Subsequently, we constructed a prognostic model and demonstrated that high risk score was significantly related to a poor survival rate. A predictive nomogram demonstrated that the nomogram model was effective prediction tool that can accurately predict the survival rate of patients with different clinical characteristics. Additionally, DAP3 expression significantly increased in both tissue samples and cell lines. Elevated levels of DAP3 were correlated with larger tumor size and higher alpha-fetoprotein (AFP) levels, and Cox analysis confirmed that DAP3 was a clinically independent prognostic marker. Finally, cell assays revealed that the knockdown of DAP3 significantly impeded cell proliferation and metabolic activity and induced apoptosis. Conversely, the overexpression of DAP3 had opposite effects on these cellular processes.

**Conclusions:**

Our study on DAP3 can provide a reference for HCC diagnosis, treatment and prognosis assessment.

## Introduction

Liver cancer is recognized as the leading malignancy in the digestive system, characterized by a notably high mortality rate (1). The genetic, metabolic, and inflammatory heterogeneity of liver cancer presents significant challenges in the development of effective therapies. While chemotherapy is utilized, it typically results in only modest increases in overall survival and limited improvements in quality of life (2, 3). Hepatocellular carcinoma (HCC) is the most frequent subtype of primary liver cancer and one of the leading causes of cancer-related death worldwide (4). Despite the widespread clinical application of associated biomarkers, such as α-fetoprotein (AFP), for the diagnosis of hepatocellular carcinoma (HCC), their use remains controversial and constrained by limitations (5). Thus, it is essential to clarify the underlying mechanisms of hepatocellular carcinoma (HCC) to discover novel biomarkers for early detection, prognosis, and treatment.

Mitochondria are crucial in regulating cellular life and death processes, influencing the initiation of apoptosis as well as survival, proliferation, and metabolism (6). The human mitochondrial ribosome is one of the most protein-rich ribosomes, as reported, MRP genes were significantly upregulated in HCC tumor samples and showed promising diagnostic value (7, 8). DAP3 is the only GTP-binding protein component of the small subunit of mammalian and yeast mitochondrial ribosomes and plays an important role in tumor progression (9–11).DAP3 has been shown to have dual roles in tumorigenesis, functioning either as a tumor suppressor or promoter, depending on the particular cell type and tumor context. These findings suggest that DAP3 may have distinct biological functions across various cancers. For example, DAP3 is overexpressed in several cancer types, including human pancreatic cancer, invasive glioblastoma multiforme, and human thyroid tumors (12–15). Conversely, decreased expression of DAP3 has been observed in gastric cancer and colorectal cancer (CRC) (16, 17). Furthermore, DAP3 expression is correlated with tumor stage and clinical outcomes in breast cancer patients (18). Notably, downregulation of DAP3 has been shown to reduce mitochondrial respiration in HeLa cells, underscoring the essential role of DAP3 in mammalian cells (11). In conclusion, the potential roles of DAP3 in carcinogenesis and cancer development warrant further investigation. Although previous studies have reported on DAP3 expression and its basic functions in liver cancer (19), this study provides new insights from the perspective of the immune microenvironment and prognostic modeling, thereby expanding the functional understanding of DAP3 within the tumor microenvironment. These phenotypic data will further enhance our understanding of the potential biological roles of DAP3.

In this study, we systematically investigated the functions of DAP3 by integrating bioinformatics analyses with experimental approaches. Using public datasets, we performed survival analysis, Gene Set Enrichment Analysis (GSEA), Kyoto Encyclopedia of Genes and Genomes (KEGG) analysis, immune infiltration analysis, single-cell sequencing analysis, mutation analysis, and chemotherapeutic efficacy analysis. From these studies, we constructed a risk score prognostic model and a nomogram for prediction. *In vitro* findings confirmed the overexpression of DAP3, and further investigations demonstrated that DAP3 regulates cell proliferation, apoptosis, cell cycle dynamics, and metastasis. Thus, DAP3 may hold significant promise for cancer prognosis and treatment.

## Materials and methods

### Clinical samples

HCC tissues were obtained from Nantong Third Hospital Affiliated with Nantong University. This study received approval from the ethics committees of Nantong Third Hospital Affiliated with Nantong University, and written consent was obtained from all patients or their guardians. None of the patients had undergone radiotherapy or chemotherapy prior to surgery.

### Data acquisition and analysis

We downloaded expression profile data and clinical phenotype data for liver hepatocellular carcinoma (LIHC) from the TCGA database (https://www.cancer.gov/tcga). Additionally, we obtained single-cell sequencing data (CSE000000) from the CNGBdb database (https://db.cngb.org/). The GSE14520 dataset was acquired from the GEO database (https://www.ncbi.nlm.nih.gov/), while the LIRI-JP sequencing data were sourced from the ICGC database (https://dcc.icgc.org/). Furthermore, we downloaded sequencing data for 33 types of pan-cancer along with the corresponding survival information from UCSC Xena (https://xena.ucsc.edu/).

### Enrichment analysis

In the TCGA cohort, we conducted differential analysis on tumor and adjacent normal tissues, as well as on groups stratified by DAP3 expression levels. We identified consistently upregulated and downregulated genes as characteristic markers for the DAP3-stratified groups. Next, we conducted Gene Ontology (GO) and Kyoto Encyclopedia of Genes and Genomes (KEGG) analyses on the candidate genes using the clusterProfiler R package. Additionally, we downloaded gene sets related to the hall v2023.1.Hs.symbols pathways from the GSEA website and conducted Gene Set Enrichment Analysis (GSEA) to identify significantly different biological processes between the low-expression and high-expression groups by ranking the changes in gene expression.

### Genomic alterations and mutation profiles

To explore the relationship between mutations and DAP3 expression levels, we downloaded somatic SNP data for liver cancer from TCGA via UCSC Xena. We analyzed mutation differences between groups using the maftools package and conducted statistical tests on the calculated tumor mutational burden. Additionally, we calculated the frequency of copy number variations (CNVs) and performed online analysis using GISTIC2.

### Immune infiltration analysis

We estimated immune scores using the TIMER, CIBERSORT, and MCPcounter algorithms and predicted potential responses to immune checkpoint inhibitors in HCC using the Tumor Immune Dysfunction and Exclusion (TIDE) method. The proportion of responders among different sample types was illustrated using the ggplot2 package. Additionally, we generated box plots and incorporated immune checkpoint-related genes and utilized the Wilcoxon test to assess the significance of differences between different groups.

### Construction of a prognostic risk score model for HCC

We used GSE144269, GSE14520, LIRI_JP, and OEP000321 as validation sets, with TCGA as the training set. We identified 73 prognostic genes through univariate Cox regression (p < 0.05) in the DAP3-stratified groups and validated 33 genes that appeared more than 500 times through 80% resampling. Using the Bioinformatics Soup database, we applied these 33 genes in the LIHC cohort to create a machine learning model with leave-one-out cross-validation (LOOCV) and calculated the C-index for each model. HCC patients were categorized into high-risk and low-risk groups according to the median risk score, and the predictive ability was assessed using the area under the time-dependent receiver operating characteristic curve (AUROC). Risk curves were plotted to assess survival differences between groups.

### Construction and validation of predictive models for clinical trials

We performed univariate and multivariate analyses using TCGA data. We also conducted decision curve analysis (DCA) to assess the net benefit at different risk thresholds, evaluating the clinical applicability of the risk score model. Next, we constructed a nomogram that integrated clinical characteristics using the ‘regplot’ package for survival prediction. Calibration and ROC curves were plotted to evaluate the predictive accuracy of the nomogram.

### Chemotherapy drug sensitivity analysis

Based on CTRP (Cancer Therapeutics Response Portal, https://portals.broadinstitute.org/ctrp) and PRISM (Profiling Relative Inhibition Simultaneously in Mixtures, https://depmap.org/portal/prism/) datasets, we conducted a drug sensitivity assay, analyzed the differences in AUC values for each chemotherapy drug among the high- and low-risk score groups and performed correlation analysis.

### Cell lines and cell culture

LO2 and five HCC cell lines (PLC/PRF/5, HCCLM3, SMMC-7721, SK-Hep-1, and Li-7) were obtained from the Cell Culture Facility (Shanghai, China). PLC/PRF/5 and SK-Hep-1 cells were cultured in MEM (Gibco, USA) supplemented with 10% fetal bovine serum (Gibco, USA). LO2 and SMMC-7721 cells were cultured in RPMI-1640 medium (Gibco, USA) supplemented with 10% FBS, and HCCLM3 cells were cultured in DMEM (Gibco, USA) supplemented with 10% FBS at 37°C with 5% CO2.

### Construction of plasmids and siRNAs

The siRNAs targeting the negative controls si-NC, si-1, and si-3 were purchased from Gene Pharma (Suzhou, China). The pcDNA3.1-DAP3 for the overexpression of DAP3 and the empty plasmid (pc-DNA3.1-NC) were purchased from Gene Pharma (Suzhou, China). We used Lipofectamine3000 (Invitrogen, USA) for transfection and overexpression according to the manufacturer’s instructions.

si-1 sense: 5’-GGAUGGAAUCAAUGCUCUUTT-3’;
antisense: 5’-AAGAGCAUUGAUUCCAUCCTT-3’
si-3 sense: 5’ -CCCUAAGUCUUUGCCAUGUTT-3’;
antisense: 5’-ACAUGGCAAAGACUUAGGGTT -3’

### IHC staining

Dewaxing and rehydration were performed in graded ethanol, followed by citrate antigen retrieval. The tissues were sealed at room temperature and incubated overnight at 4°C with an anti-DAP3 antibody (Santa Cruz, 1:200). The sections were then incubated with a horseradish peroxidase-conjugated secondary antibody at room temperature for 30 minutes. Staining was performed using 3,3′-diaminobenzidine (DAB), followed by washing and counterstaining with hematoxylin. Immunohistochemical scoring was based on the product of the staining intensity and extent scores. Staining intensity: 0 (no staining), 1 (weak staining), 2 (moderate staining), and 3 (strong staining); staining extent scoring: 0 (≤10%), 1 (10%-25%), 2 (25%-50%), 3 (50%-75%), or 4 (75%).

### RNA preparation and RT-qPCR

Total RNA was isolated with TRIzol reagent (Invitrogen, USA), reverse transcribed into cDNA with a Revert Aid First Strand cDNA Synthesis Kit (Thermo Fisher Scientific, USA), and finally, SYBR reagent (Bio-Rad, China) and specific primers (Generay, China) were used for quantification. After normalization to GAPDH, the relative expression was calculated. The RT–qPCR sequences of primers used were as follows:

GAPDH forward: 5’-GGACCTGACCTGCCGTCTAG-3’,
GAPDH reverse: 5’-GTAGCCCAGGATGCCCTTGA-3’;
DAP3 forward: 5’-TCCAGCTACAACAAACAGCG-3’,
DAP3 reverse: 5’-CTCACCCGTGTTATGCCCTG-3’.

### Western blot

Total protein was extracted in RIPA buffer with 1% PMSF (Beyotime, China). The samples were separated using 4–12% SDS-PAGE gels and subsequently transferred to polyvinylidene fluoride (PVDF) membranes (Millipore, USA). After blocking, the membranes were incubated with primary and secondary antibodies, and protein expression was detected using an enhanced chemiluminescence (ECL) kit (Tanon, China). The antibodies used for Western blot were as follows: anti-GAPDH antibody (1:20000, Proteintech, China), anti-DAP3 antibody (1:200, Santa Cruz, China), HRP-conjugated-β-actin antibody (1:5000, Proteintech, China) and HRP-conjugated goat anti-mouse IgG antibody (1:1000, Beyotime, China).

### Flow cytometry

The transfected cells were harvested for apoptosis analysis, and an Annexin V/7-AAD kit (BD Biosciences, USA) was used to detect the apoptotic rate under the constructions, with analysis performed using FlowJo.

### CCK-8 assay

A total of 3,000 transfected cells were seeded in 96-well plates and incubated overnight. The cells were subsequently incubated with CCK-8 solution (MCE, USA) for 2 hours, and the optical density (OD) value was subsequently measured at 450 nm using a plate reader (Thermo, USA) every 24 hours.

### Colony formation assay

A total of 2000 transfected cells were seeded in a six-well plate. After 2 weeks, the cells were fixed with 4% formaldehyde (Beyotime, China) and stained with 0.1% crystal violet (Sigma, USA). Cell colonies were counted and analyzed using GraphPad Prism 8.0.

### Transwell assay

Transwell assays were conducted using the concentration difference of serum. The chamber (Costar, USA) and Matrigel (BD, USA) were used for invasion assays, whereas migration assays were performed with no gel. After 48 hours, the cells were fixed with 4% paraformaldehyde (Beyotime, China), stained with 0.1% crystal violet (Sigma, USA), and photographed under a microscope (Olympus, Japan). The cells in random fields were counted and analyzed using GraphPad Prism 8.0.

### Co expression analysis

LinkedOmics (http://www.linkedomics.org/login.php) platform include Linkfinder and LinkInterpreter section. Linkfinder was used to obtain co-expressed proteins associated with DAP3 in HCC.

### Statistics analyses

The data are presented as the means ± standard deviations (SDs). A t test was used for comparisons between two groups, whereas one-way analysis of variance (ANOVA) was used for comparisons involving more than two groups. Statistical significance was calculated using GraphPad Prism 8.0 and SPSS 17.0. All experiments were repeated independently at least twice. A p value of less than 0.05 was considered statistically significant.

## Results

### DAP3 expression was related to poor prognosis in public datasets

**Figure 1:**
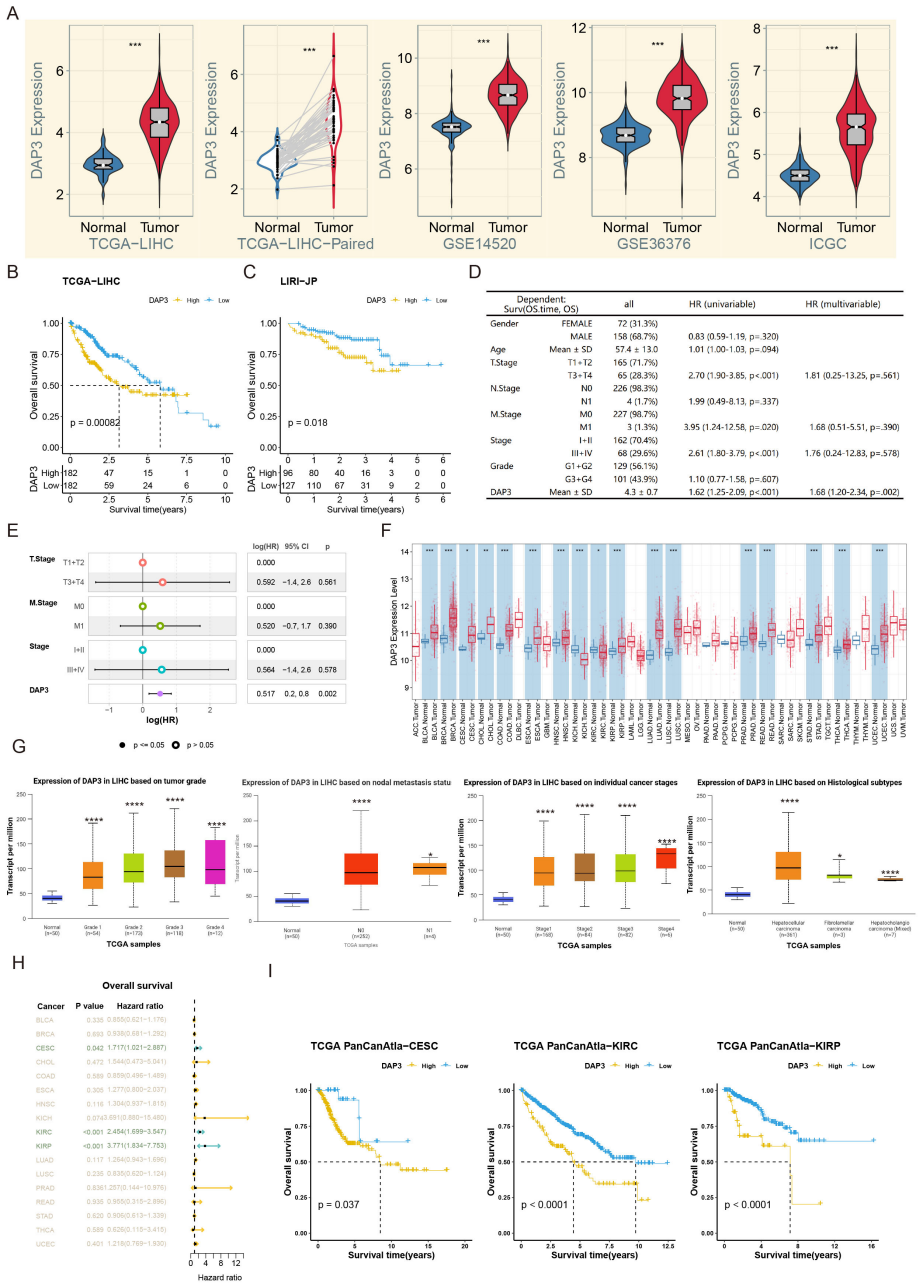
DAP3 expression was related with poor prognosis in public datasets: **(A)** Relative DAP3 expression levels across TCGA, GEO, and ICGC datasets. **(B)** Survival curves derived from TCGA datasets. **(C)** Survival curves based on ICGC datasets. **(D)** Univariate Cox regression analysis for overall survival in HCC. **(E)** Multivariate Cox regression analysis related to overall survival in HCC. **(F)** DAP3 expression in various pan-cancers using TIMER2.0 datasets. **(G)** DAP3 expression in various HCC subtypes using UALCAN datasets. **(H)** Overall survival analysis across pan-cancers. **(I)** Survival curves in CESC, KIRC, and KIRP. *P<0.05, **P<0.01, ***P<0.001, ****P < 0.0001.

We analyzed DAP3 expression using datasets from TCGA, ICGC, and GSE14520. Our results revealed significant upregulation of DAP3 expression in tumor samples (**Figure 1A**). Further assessment of the prognostic value of DAP3 in TCGA and ICGC cohorts revealed that higher DAP3 expression was significantly associated with shorter overall survival (**Figures 1B, C**). Univariate Cox regression analysis identified DAP3 as a risk factor for poor prognosis, yielding a hazard ratio (HR) of 1.62 (**Figure 1D**). Multivariate analysis confirmed that DAP3 served as an independent prognostic factor (**Figure 1E**). Additionally, DAP3 was found to be broadly overexpressed across various cancers, and linked to the severity of HCC lesions (**Figures 1F, G**). High expression was significantly correlated with poor prognosis in multiple cancer types, such as cervical squamous cell carcinoma (CESC), kidney renal clear cell carcinoma (KIRC), and kidney renal papillary cell carcinoma (KIRP) (**Figures 1H, I**).

### Kyoto encyclopedia of genes and genomes analysis and GSEA

**Figure 2:**
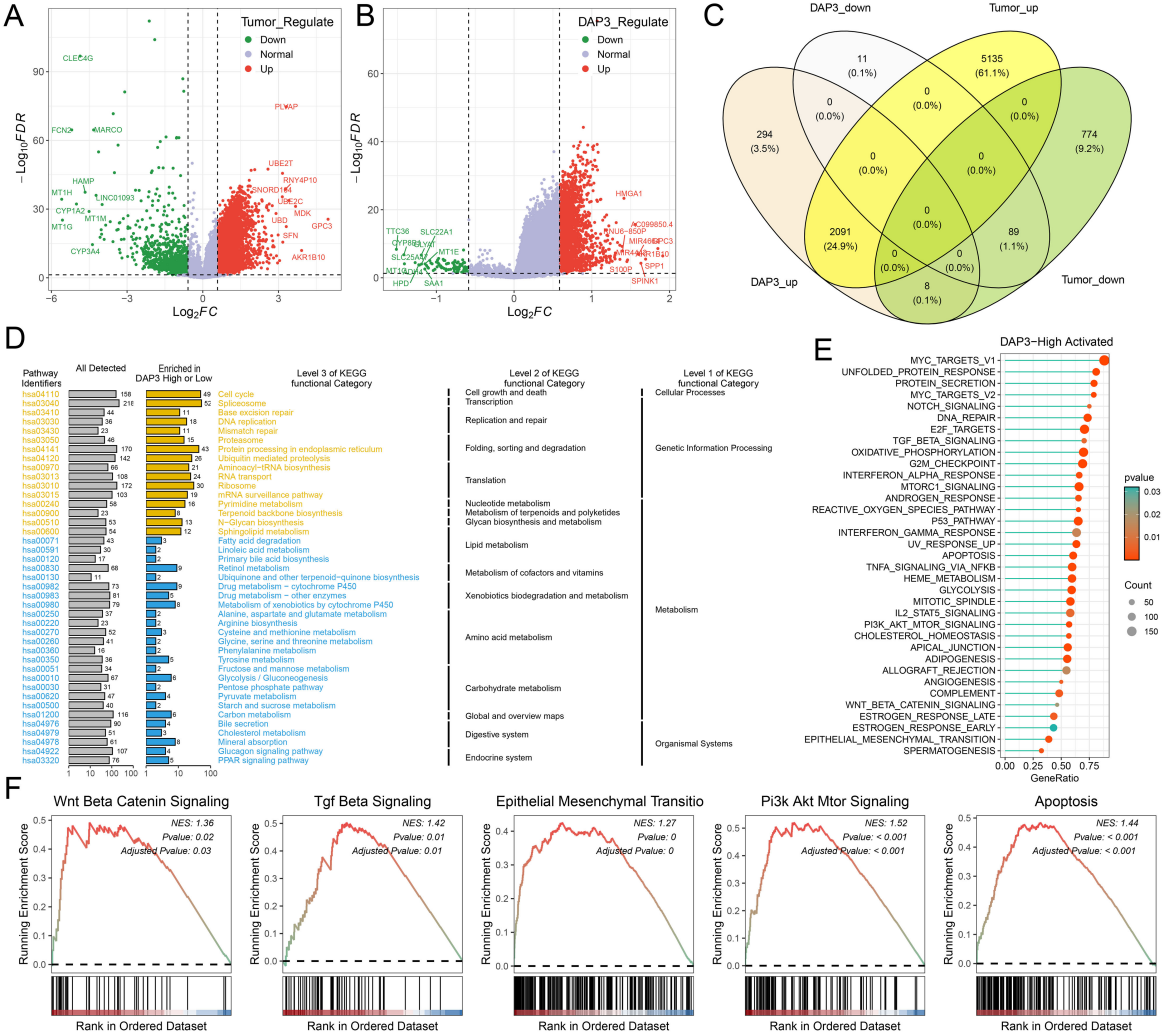
Kyoto Encyclopedia of Genes and Genomes (KEGG) and GSEA analysis: **(A)** A volcano plot illustrating differential gene expression between tumor-adjacent groups. **(B)** A volcano plot depicting differential gene expression between high and low expression groups. **(C)** A Venn diagram showcasing the overlap of differentially expressed genes between the two comparisons. **(D, E)** KEGG analysis highlighting enriched pathways in the high expression groups. **(F)** GSEA analysis demonstrating enriched signaling pathways.

In the TCGA cohort, we conducted differential analysis between the tumor-adjacent group and the high-low-expression group, followed by the generation of a volcano plot to visualize the results (**Figures 2A, B**). A Venn diagram illustrated the extensive overlap of differentially expressed genes between the two groups (**Figure 2C**). Next, we performed KEGG enrichment analysis, which revealed distinct pathways enriched in the high-DAP3 group versus the low-DAP3 group, such as those related to cell metabolism, transcription, drug metabolism, and the immune environment, providing new avenues for future research. (**Figures 2D, E**). GSEA revealed that several cancer-related pathways, including the Wnt, apoptosis, TGF-beta, epithelial-mesenchymal transition, and PI3K pathways, were enriched in the high-DAP3 group (**Figure 2F**). These findings indicate that DAP3 may play a major role in regulating cell proliferation and migration.

### Correlation between DAP3 and immune-related functions in HCC

**Figure 3:**
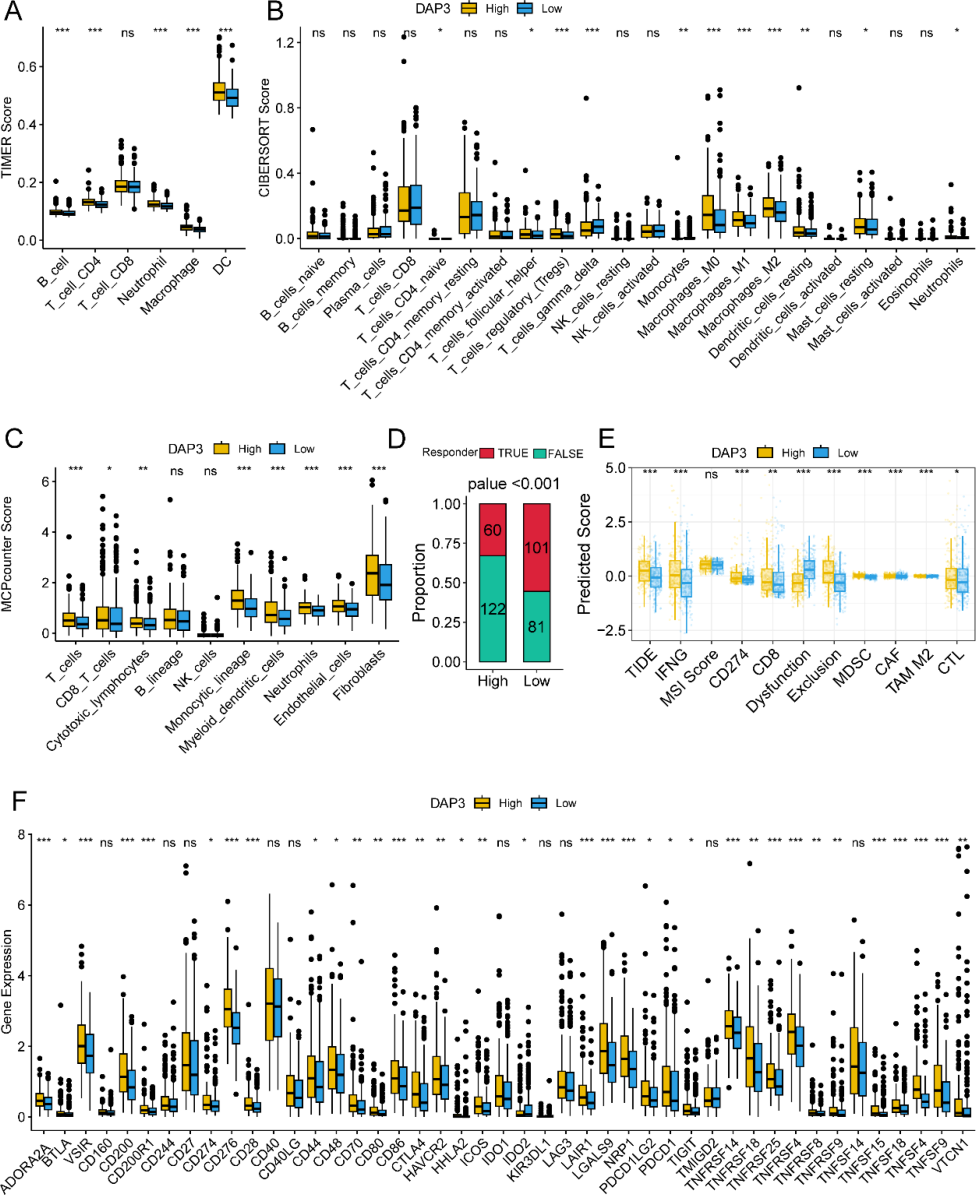
Correlation between DAP3 and immune-related functions in HCC: **(A)** Immune cell infiltration in low and high DAP3 expression groups based on TIMER scores. **(B)** Immune cell infiltration in low and high DAP3 expression groups based on CIBERSORT scores. **(C)** Immune cell infiltration in low and high DAP3 expression groups based on MCP-counter scores. **(D)** Proportional plot illustrating the relationship between DAP3 expression and responder numbers based on TIDE scores. **(E)** Predicted immune response and tumor microenvironment characteristics in low and high DAP3 expression groups. **(F)** Expression levels of immune checkpoint genes in low and high-risk groups. *P<0.05,**P<0.01,***P<0.001.

First, we calculated the scores of six immune cell types using the TIMER, CIBERSORT, and MCP counter algorithms. The results indicated that patients in the high DAP3 expression group had higher immune cell scores across most immune cell types (**Figures 3A–C**). To evaluate patient responsiveness to immunotherapy, we utilized TIDE data for prediction, which revealed that patients with high DAP3 expression had greater potential for immune evasion (**Figure 3D**). Additionally, we found that the high DAP3 expression group showed a significantly higher response to Sorafenib treatment compared to the low DAP3 expression group, with a statistically significant difference (**Supplementary Figure 1A**). The ROC curve further validated the sensitivity and specificity of DAP3 in predicting treatment response (**Supplementary Figure 1B**). Moreover, we observed significant differences in the predicted scores for features such as TIDE, dysfunction, exclusion, and myeloid-derived suppressor cells (MDSCs) based on DAP3 expression levels (**Figure 3E**). Furthermore, analysis of immune checkpoint genes (20) identified in previous studies revealed higher expression levels of these genes in the high DAP3 group (**Figure 3F**). These findings showed that DAP3 could influence the tumor microenvironment and may be an important biomarker for immunotherapy.

### Potential role of DAP3 in HCC genomic alterations

**Figure 4:**
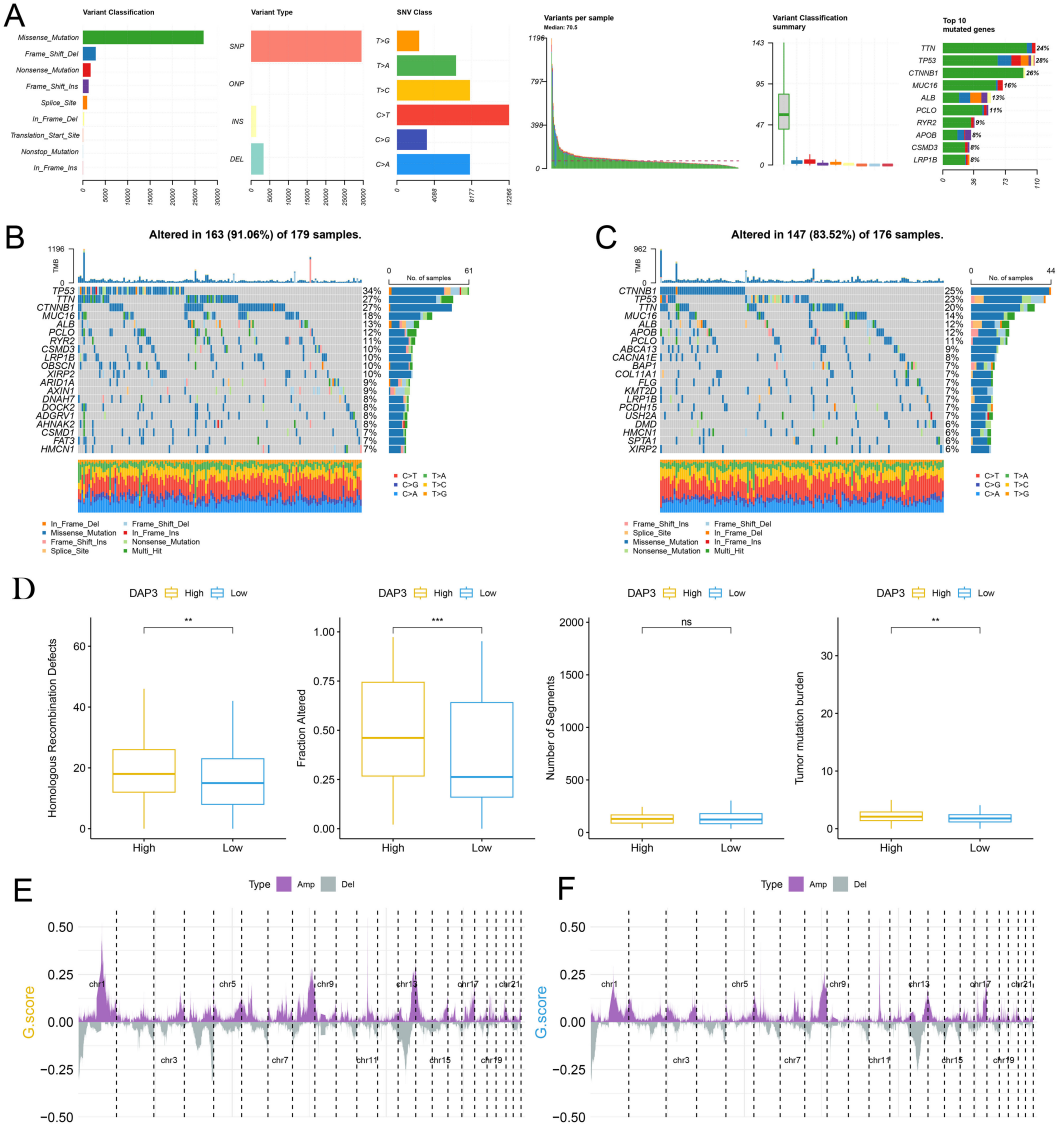
The potential role of DAP3 in HCC genomic alterations: **(A)** Classification and frequency of gene mutations. **(B)** Waterfall plot depicting gene mutations in the high-DAP3 expression group. **(C)** Waterfall plot illustrating gene mutations in the low-DAP3 expression group. **(D)** DAP3 expression levels and genomic alterations in low versus high DAP3 expression groups. **(E)** Distribution of copy number variations (CNVs) across different chromosomal regions in the high-DAP3 expression group. **(F)** Distribution of CNVs across different chromosomal regions in the low-DAP3 expression group. **P<0.01, ***P<0.001.

To investigate the relationship between mutations and DAP3 expression levels, we downloaded somatic SNP data for HCC from the TCGA database via UCSC Xena. Our analysis revealed that the predominant mutation type was missense mutation, with single nucleotide polymorphisms (SNPs) being the primary variant type in hepatocellular carcinoma (**Figure 4A**). We subsequently examined the distribution of gene mutations in samples with high DAP3 expression (**Figure 4B**) compared with those with low DAP3 expression (**Figure 4C**). The box plots indicated that the high DAP3 expression group exhibited significantly more homologous recombination defects and fusion genes than the low DAP3 expression group. Additionally, there was a notable difference in tumor mutation burden, suggesting that DAP3 expression levels may be associated with genomic instability and mutation rates (**Figure 4D**). Furthermore, gene amplifications and deletions were widespread across various chromosomes (**Figures 4E, F**). These findings showed that high DAP3 expression may be linked to increased mutation rates, homologous recombination defects, and copy number variations, indicating a potential role for DAP3 in tumor genomic alterations.

### A single-cell expression atlas and identification of DAP3 in HCC by scRNA-seq

**Figure 5:**
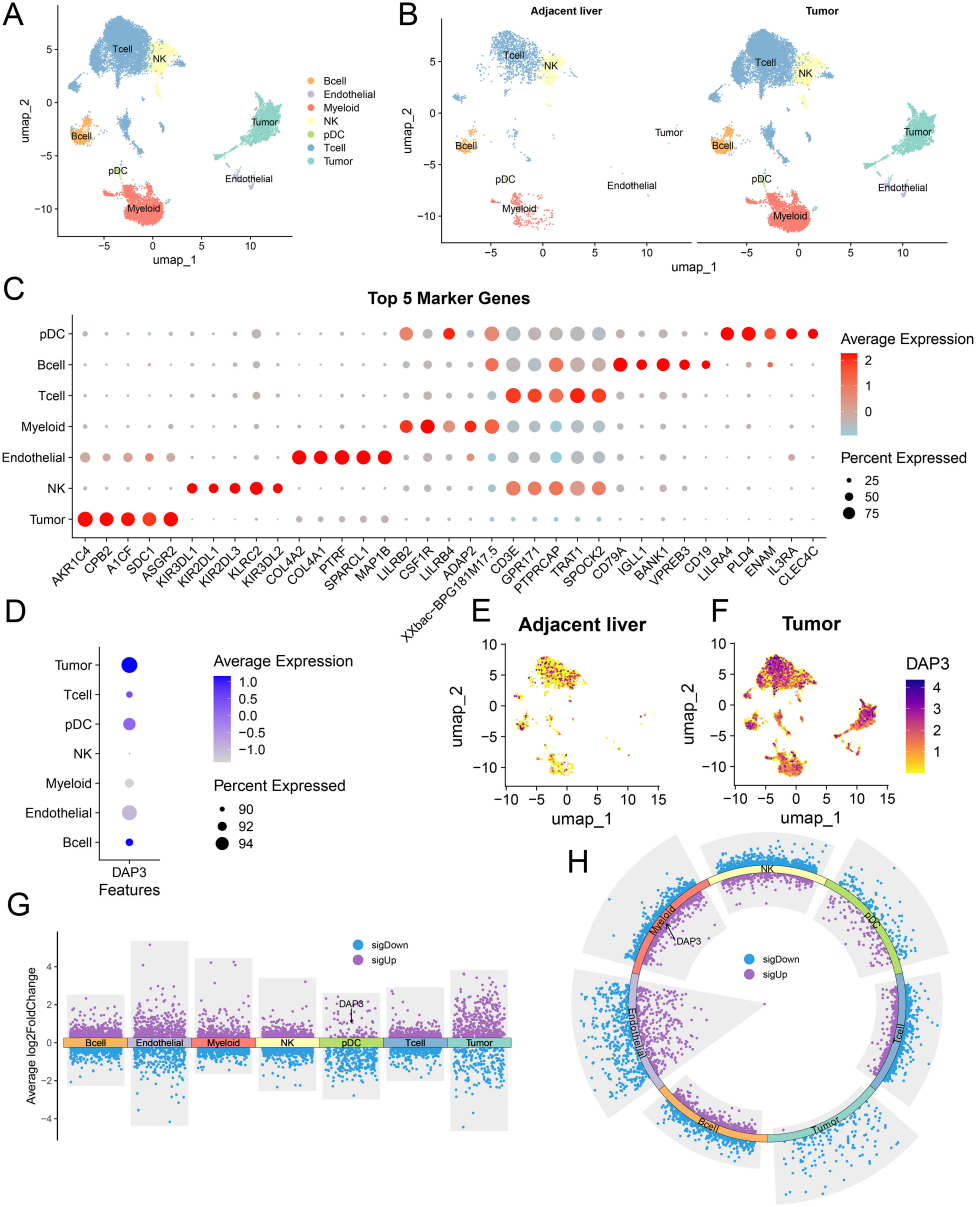
A single-cell expression atlas and the identification of DAP3 in HCC by scRNA-seq: **(A)** Distribution of various cell types in hepatocellular carcinoma (HCC). **(B)** Comparison of cell type distribution in adjacent liver and tumor tissues. **(C)** Top five marker genes for different cell types. **(D)** Expression levels of DAP3 across various cell types. **(E)** Expression and distribution of DAP3 in adjacent liver tissues. **(F)** Expression and distribution of DAP3 in tumor tissues. **(G)** Comparison of gene expression levels in different cell types within tumor tissues versus adjacent tissues. **(H)** Gene expression levels in different cell types during recurrence compared to non-recurrence conditions.

Stemness scores (CytoTRACE2) were significantly higher in the high DAP3 expression group (DAP3+) compared to the low expression group (DAP3−) (**Supplementary Figure 2A**), and a clear positive correlation was observed between DAP3 expression levels and CytoTRACE2 scores (**Supplementary Figure 2B**).The UMAP distribution further revealed heterogeneity in stemness levels across different cell populations (**Supplementary Figure 2C**), suggesting a potential role for DAP3 in regulating cellular stemness. To further investigate the distribution characteristics of DAP3 in the tumor microenvironment, we analyzed the relationships and gene characteristics among distinct cell populations through the integration of single-cell transcriptome data. Using a combination of classical markers, we identified seven major cell groups (**Figure 5A**). We compared the distribution of cells between adjacent and tumor samples, revealing significant differences in cell distribution between tumor tissue and adjacent normal liver tissue (**Figure 5B**). Next, we identified the top five most significant marker genes for each subcluster (**Figure 5C**). DAP3 was widely expressed in tumor cells, plasmacytoid dendritic cells (pDCs), and endothelial cells, with higher expression levels in tumor samples than in adjacent samples (**Figures 5D–F**). Differential analysis between tumor and adjacent samples revealed that DAP3 was upregulated in pDCs (**Figure 5G**). Furthermore, differential analysis between primary and recurrent samples indicated significant upregulation of DAP3 in myeloid cells, suggesting that DAP3 may influence tumor development and progression (**Figure 5H**). Finally, we conducted differential gene expression and functional enrichment analyses, which revealed that the differentially expressed genes in the DAP3+ group were significantly enriched in lipid metabolism, glycolysis, and the PPAR signaling pathway (**Supplementary Figures 2D, E**). Metabolic heatmap analysis further demonstrated substantial activation of lipid metabolism, glycolysis, and oxidative phosphorylation in the DAP3+ group, particularly in recurrent tumor samples (**Supplementary Figure 2F**). These findings suggest that DAP3 may drive metabolic reprogramming, thereby promoting rapid tumor cell proliferation and invasion.

### Construction and evaluation of prognostic risk-score model

**Figure 6:**
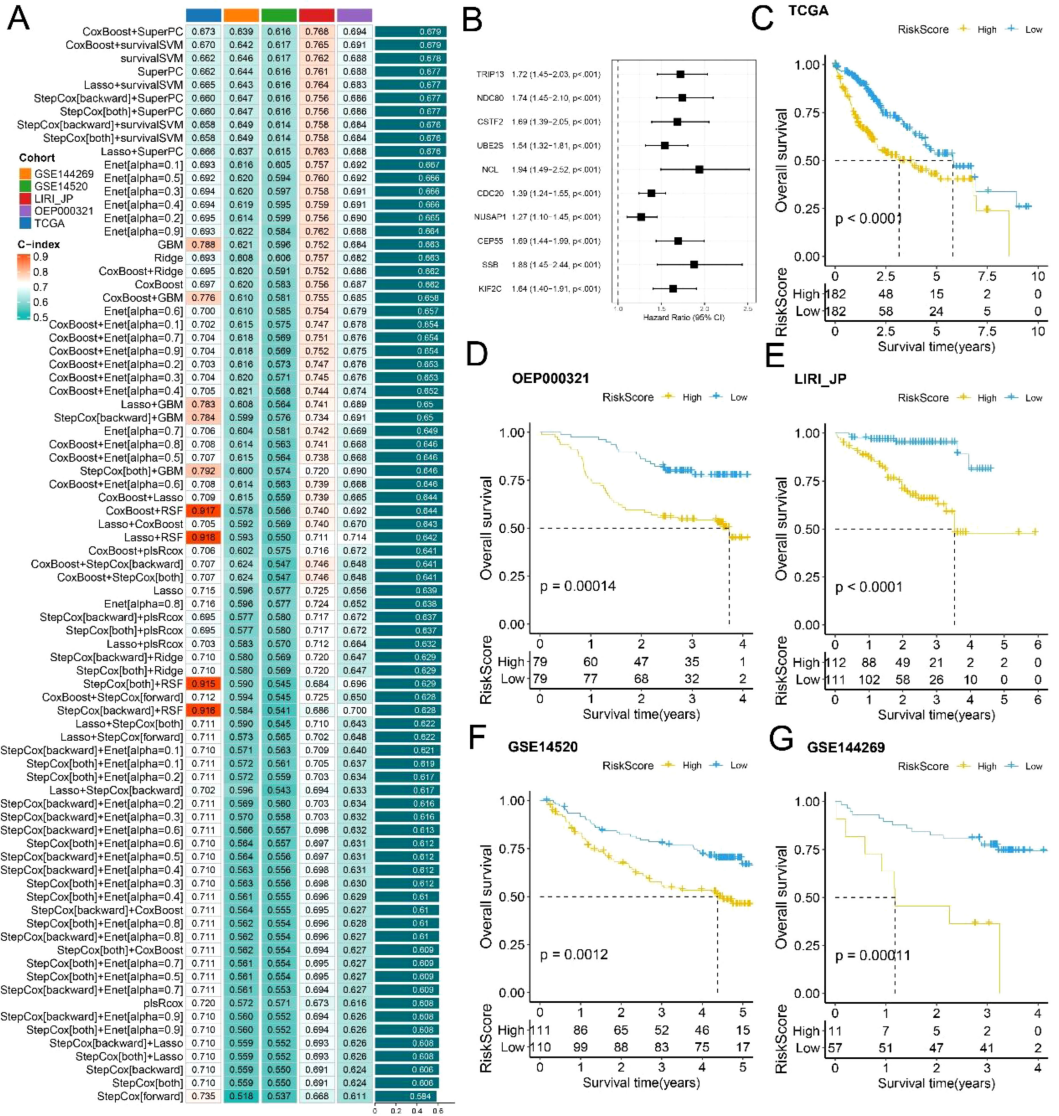
Construction and Evaluation of Prognostic Risk-Score Model: **(A)** Performance comparison of survival prediction models across different cohorts, assessed using C-index. **(B)** Forest plot highlighting 10 key genes. **(C-G)** Survival curves comparing low- and high-risk score groups based on the TCGA, OEP000321, LIRI_JP, GSE14520, and GSE144269 datasets.

Through a leave-one-out cross-validation (LOOCV) framework, we fitted 101 predictive models and calculated the concordance index (c-index) for each model across all the validation datasets. The optimal model, a combination of Cox Boost and Super PC, achieved the highest average c-index in the test set (**Figure 6A**). Additionally, we identified 10 key genes (TRIP13, NDC80, CSTF2, UBE2S, NCL, CDC20, NUSAP1, CEP55, SSB, and KIF2C), as illustrated in the forest plot (**Figure 6B**). The risk scores for both the training and validation sets indicated that patients in the high-risk group had poorer prognoses, whereas those in the low-risk group exhibited better prognoses across all cohorts (**Figures 6C–G**). Furthermore, time-dependent ROC curve analysis was used to assess the predictive efficacy of the prognostic signature based on both training and validation datasets (**Supplementary Figure 3**). The risk score demonstrated strong predictive ability across different liver cancer datasets, with higher risk scores significantly associated with poorer survival outcomes.

### Integration of the risk score with clinicopathological features

**Figure 7:**
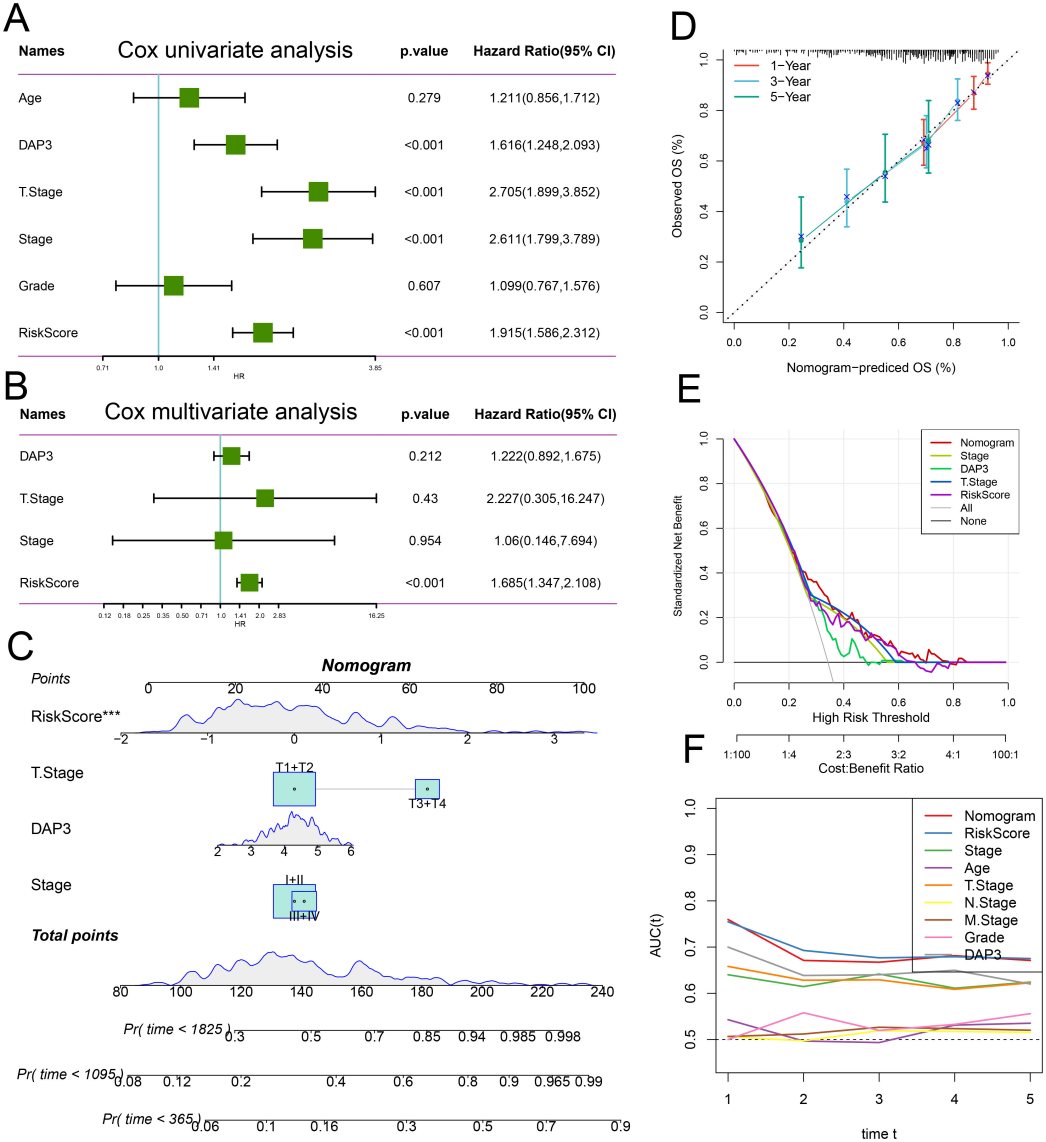
Integration of risk score with clinicopathological features: **(A)** Univariate Cox regression analysis of the risk score alongside other clinical features. **(B)** Multivariate Cox regression analysis incorporating the risk score and additional clinical characteristics. **(C)** Nomogram designed to predict patients’ survival probabilities at various time intervals. **(D)** Calibration curves illustrating the predictions of the established nomogram for 1-, 3-, and 5-year overall survival. **(E)** Decision curve analysis for 5-year overall survival in the TCGA-LIHC dataset. **(F)** Time-dependent ROC curve analysis to assess the predictive efficacy of the prognostic signature.

Next, we conducted univariate and multivariate Cox regression analyses incorporating risk scores and clinical characteristics, revealing that the risk score was the most significant prognostic factor (**Figures 7A, B**). To quantify patients’ risk assessments and survival probabilities, we developed a nomogram that integrated the risk score with other clinicopathological features. The results demonstrated that the risk score had the most significant impact on survival prediction (**Figure 7C**). We further evaluated the predictive accuracy of the model using calibration curves. The calibration curves at 1, 3, and 5 years closely aligned with the standard curve, indicating that the nomogram demonstrated good predictive performance (**Figure 7D**). Additionally, we employed DCA to assess the model’s reliability, which revealed that both the risk score and the nomogram provided significantly greater net benefits than the extreme curves (**Figure 7E**). Time-dependent AUC analysis illustrated the predictive ability of the different models over time, with the nomogram and risk score performing well at multiple time points (**Figure 7F**). Thus, the risk score and nomogram model serve as effective predictive tools that, in conjunction with clinical characteristics, can accurately predict patient survival.

### Identification of potential therapeutic agents for high-risk HCC patients

**Figure 8:**
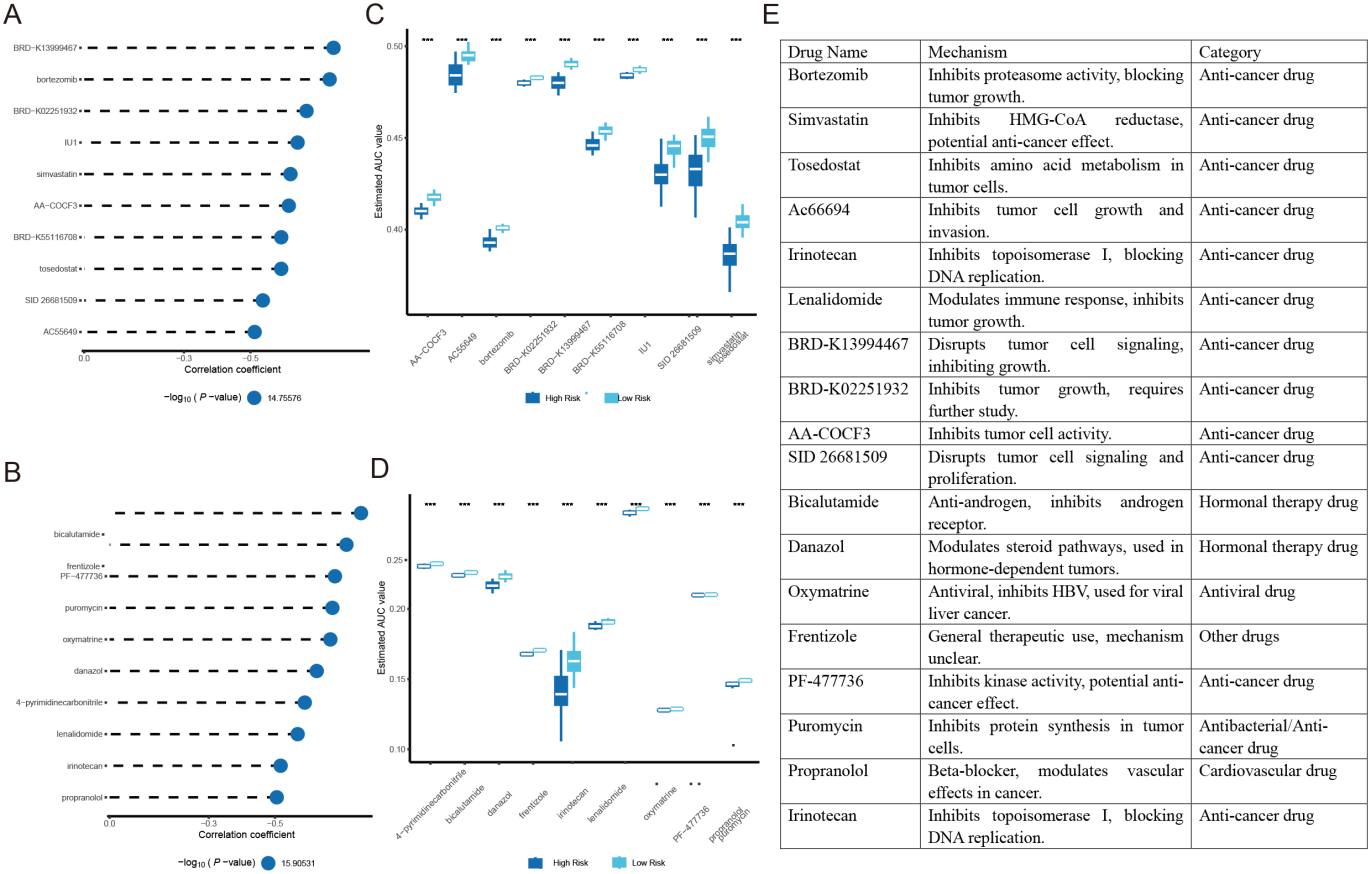
Identification of potential therapeutic agents for high-risk HCC patients: **(A)** Spearman correlation analysis of drugs derived from CTRP. **(B)** Spearman correlation analysis of drugs derived from PRISM. **(C)** Analysis of differences AUC values for drugs derived from CTRP. **(D)** Analysis of differences in response (AUC values) for drugs derived from PRISM. **(E)** Screening and Mechanistic Classification of drugs. ***P<0.001.

The CTRP and PRISM drug databases were utilized to identify potential therapeutic agents. Differential drug response analysis between the high-risk and low-risk score groups was performed, identifying drugs with lower predicted AUC values in the high-risk score group. Subsequently, Spearman correlation analysis between the AUC values and risk scores was conducted to select drugs with a negative correlation coefficient (**Figures 8A, B**). All drugs exhibited lower estimated AUC values in the high-risk score group than in the low-risk score group, indicating increased drug sensitivity (**Figures 8C, D**). The mechanisms of potential drugs were searched from Selleck websites (**Figure 8E**).

### Increased DAP3 expression was related to poor prognosis in HCC patients

**Figure 9:**
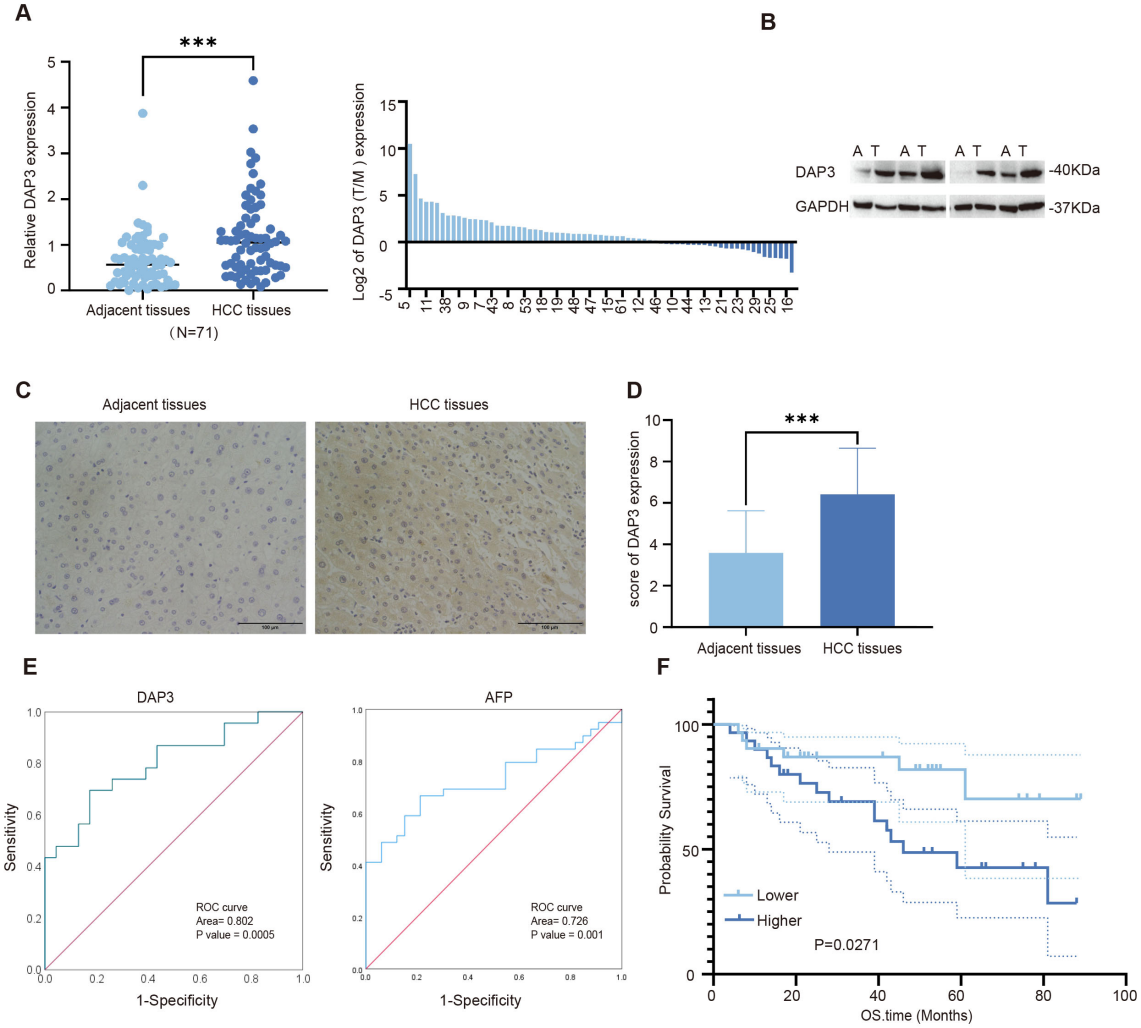
Increased DAP3 expression was associated with poor prognosis in HCC patients: **(A)** Relative DAP3 expression levels in HCC tissues as measured by qRT-PCR (n = 71). **(B)** DAP3 expression analyzed via Western blot in HCC tissues (n = 4). **(C)** Representative images of DAP3 expression in HCC tissues obtained through immunohistochemical staining (magnification: 40X; scale bar: 100 μm). **(D)** IHC scores for DAP3 expression. **(E)** ROC curve analysis of DAP3 expression and AFP value. **(F)** Overall survival (OS) time compared using Kaplan–Meier analysis. ***P<0.001.

Based on expression patterns from various datasets, we collected tumor and paired adjacent tissues from Nantong Third People’s Hospital. We found that DAP3 expression was significantly higher in HCC tissues than in the adjacent tissues using RT-qPCR and Western blot analyses (**Figures 9A–C**), which was also confirmed by IHC analysis (**Figure 9D**). ROC analysis revealed that the AUC of DAP3 was 0.802 (95% CI: 0.67–0.92, p<0.05), and the area of AFP was 0.726 (95% CI: 0.608–0.845, p<0.05) (**Figure 9E**). Kaplan-Meier survival analysis revealed that patients with higher DAP3 expression had shorter overall survival (**Figure 9F**). Elevated DAP3 levels were correlated with increased AFP levels (P=0.044) and larger tumor size (P=0.024) (**Table 1**). Univariate Cox regression revealed that DAP3 expression (p=0.047) and TNM stage (p=0.044) were risk factors for HCC. Multivariable analysis confirmed that DAP3 expression (p=0.040) was an independent prognostic indicator for overall survival (OS) in HCC patients (**Table 2**). Thus, DAP3 is a promising independent prognostic marker for overall survival.

**Table 1:** Correlation of DAP3 expression with clinicopathological characteristics in HCC patients.

Clinicopathological Characteristics	DAP3 expression		P value
	Low expression	High expression	
Age (years)			0.904
≥58	17 (48.6%)	18 (50.0%)	
<58	18 (51.4%)	18 (50.0%)
Gender			0.945
Male	26 (74.3%)	27 (75.0%)	
Female	9 (25.7%)	9(25.0%)
AFP (ng/mL)			0.044
≥40	14 (40.0%)	23 (63.9%)	
<40	21 (60.0%)	13 (36.1%)
Tumor size (cm)			0.024
≥4	12 (34.3%)	22( 61.1%)	
<4	23 (65.7%)	14 (38.9%)	
Tumor stage			0.561
I-II	15 (42.9%)	13 (36.1%)	
II-IV	20 (57.1%)	23 (63.9%)	
Vascular invasion			0.259
No	24 (68.6%)	20 (55.6%)	
Yes	11 (31.4%)	16 (44.4%)	

TNM stage, tumor node-metastasis stage; AFP, alpha fetoprotein; P-value ≤ 0.05 indicate statistical significance.

**Table 2:** Univariate and multivariate Cox regression analyses related to overall survival in patients with HCC.

Index	Univariable			Multivariable		
	P value	Hazard ratio	95% Cl for Exp(B)	P value	Hazard ratio	95% Cl for Exp(B)
DAP3	0.047	2.591	1.011-6.639	0.040	2.920	1.052-8.106
Gender	0.269	0.599	0.242-1.485			
Age	0.244	1.695	0.698-4.117			
TNM Stage	0.044	2.844	1.028-7.866	0.042	2.649	1.034-6.791
AFP	0.688	0.842	0.363-1.951			
Tumor size	0.511	1.328	0.570-3.094			

P-value ≤ 0.05 indicate statistical significance.

### Knockdown of DAP3 inhibited the progression of HCC cells

**Figure 10:**
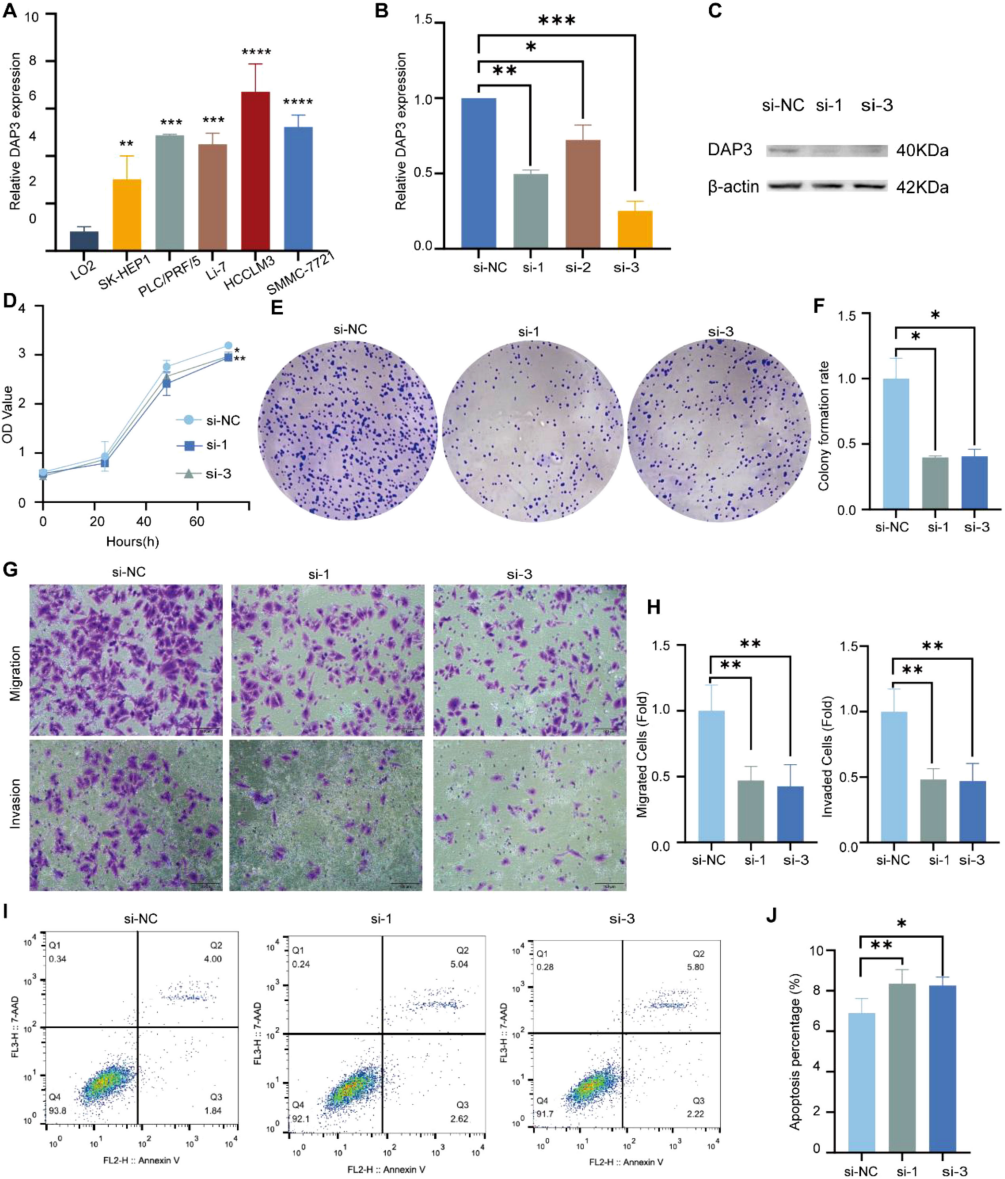
Knockdown of DAP3 inhibited the progression of HCC cells: **(A)** DAP3 expression levels in normal hepatocytes (LO2) compared to five HCC cell lines. **(B)** Knockdown efficiency in HCCLM3 cells confirmed by qRT-PCR. **(C)** Knockdown efficiency in HCCLM3 cells validated by Western blot. **(D)** CCK-8 assay results in DAP3-knockdown HCCLM3 cells. **(E)** Colony formation assay conducted in DAP3-knockdown HCCLM3 cells. **(F)** Number of colonies formed in DAP3-knockdown HCCLM3 cells. **(G)** Representative images from migration and invasion assays in DAP3-knockdown HCCLM3 cells. **(H)** Number of migrated or invaded cells in DAP3-knockdown HCCLM3 cells. **(I)** Apoptosis levels in DAP3-knockdown HCCLM3 cells. **(J)** Apoptotic rates in DAP3-knockdown HCCLM3 cells. *P<0.05,**P<0.01,***P<0.001,****P<0.0001.

DAP3 was highly expressed in HCC cell lines (**Figure 10A**). To investigate the functional role of DAP3, we transfected HCCLM3 cells with DAP3 siRNAs. RT-qPCR confirmed effective knockdown (**Figure 10B**), and Western blot analyses validated this finding (**Figure 10C**). For cell proliferation studies, CCK-8 and colony formation assays revealed that reduced DAP3 expression decreased HCCLM3 cell viability (**Figure 10D**) and significantly reduced colony numbers (**Figures 10E, F**). In transwell assays, DAP3 knockdown led to a notable decrease in the migratory and invasive capabilities of HCCLM3 cells (**Figures 10G, H**). Additionally, flow cytometric analysis revealed that DAP3 downregulation promoted apoptosis in these cells (**Figures 10I, J**).

### Overexpression of DAP3 promoted the progression of HCC cells

**Figure 11:**
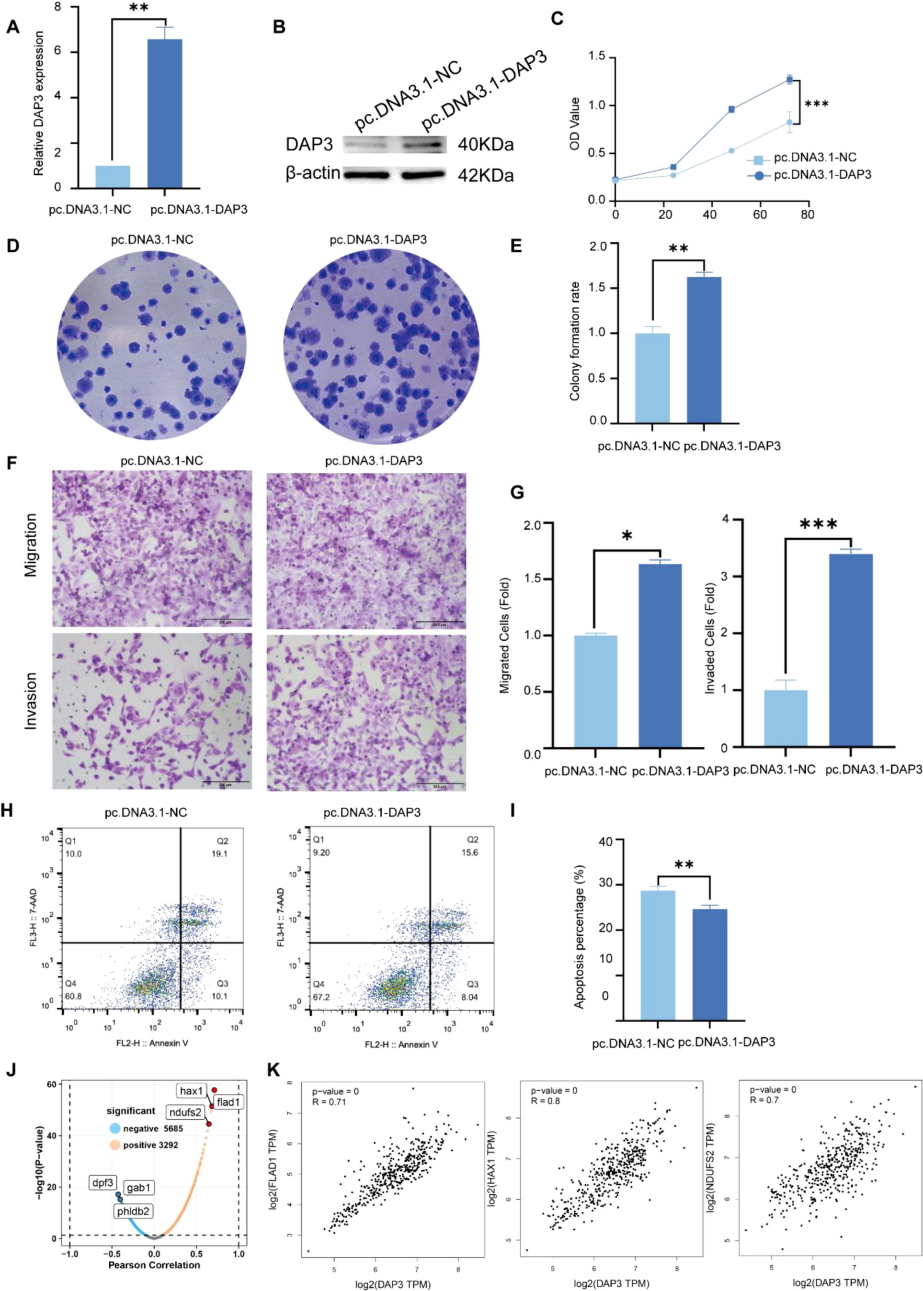
Overexpression of DAP3 promoted the progression of HCC cells: **(A)** Overexpression efficiency in SK-Hep1 cells confirmed by qRT-PCR. **(B)** Overexpression efficiency in SK-Hep1 cells validated by Western blot. **(C)** CCK-8 assay results in DAP3-overexpressing SK-Hep1 cells. **(D)** Colony formation assay conducted in DAP3-overexpressing SK-Hep1 cells. **(E)** Number of colonies formed in DAP3-overexpressing SK-Hep1 cells. **(F)** Representative images from migration and invasion assays in DAP3-overexpressing SK-Hep1 cells. **(G)** Number of migrated or invaded cells in DAP3-overexpressing SK-Hep1 cells. **(H)** Apoptosis levels in DAP3-overexpressing SK-Hep1 cells. **(I)** Apoptotic rates in DAP3-overexpressing SK-Hep1 cells. **(J)** Volcano plot shown the co-expressed protein with DAP3 in HCC. **(K)** Correlation analysis between DAP3, FLAD1, HAX1, and NDUFS2 based on GEPIA datasets. *P<0.05, **P<0.01, ***P<0.001.

To investigate the functional role of DAP3 in HCC cells, we constructed a DAP3 overexpression plasmid and transfected it into SK-Hep1 cells. Both RT-qPCR and Western blot analyses confirmed the efficient overexpression of DAP3 (**Figures 11A, B**). Subsequently, CCK-8 assays demonstrated that increased DAP3 expression significantly enhanced the viability of SK-Hep1 cells (**Figure 11C**). Furthermore, DAP3 overexpression notably increased the number of colonies formed by SK-Hep1 cells (**Figures 11D, E**). Additionally, the overexpression of DAP3 promoted both cell migration and invasion (**Figures 11F, G**). Flow cytometric analysis revealed that upregulation of DAP3 inhibited apoptosis in SK-Hep1 cells (**Figures 11H, I**). To investigate the potential function and mechanism in HCC, we constructed a co-expression network of DAP3 in HCC through the linkedomics platform. A volcano plot showing the proteins positively and negatively associated with DAP3 was constructed (**Figure 11J**), showing 5685 genes negatively correlated with DAP3 and 3292 genes exhibiting positive correlation with HCC. Correlation analysis was conducted by GEPIA datasets which revealed that mitochondrial-related genes (FLAD1, HAX1 and NDUFS2) were positively correlated with DAP3 (**Figure 11K**), suggesting that DAP3 plays an important role in OXPHOS. However, additional work on identifying the DAP3 regulatory network needs to be performed in the future.

## Discussion

Currently, cancer is widely recognized as a metabolic disorder (21). Hepatocellular carcinoma (HCC) is one of the most prevalent forms of primary liver cancer worldwide (22). HCC can be classified based on various criteria, including histological characteristics, molecular features, tumor growth patterns, etiological background, and degree of differentiation, which could help us to better understand the heterogeneity of HCC and provide guidance for precision medicine (23–25). KEGG analysis and GSEA revealed that higher DAP3 expression is linked to various biological processes, including cell metabolism, transcription, drug metabolism, and the immune environment, thereby opening new avenues for future research.

Mitochondria provide energy for eukaryotic cells by oxidizing fats and sugars to produce ATP, and oxidative phosphorylation (OXPHOS) is one of the core metabolic pathways in this process (26). As reported, DAP3 can increase mitochondrial complex I activity in HCC cells by regulating the translation and expression of MT-ND5 (19). In our study, HAX1, FLAD1 and NDUFS2, which were positively correlated with DAP3, were reported to play a crucial in mitochondria which could regulate the progression in numerous cancers (27–29), indicating that DAP3 may be an important role in OXPHOS, which could affect HCC progression. In public datasets, we concluded that DAP3 expression was linked to the subtypes of HCC, and that its high expression was linked to a poor prognosis, its elevated expression correlated with poor outcomes. Clinically, high DAP3 expression was strongly associated with larger tumor size and elevated AFP levels, demonstrating good diagnostic value according to ROC analysis. Furthermore, survival analysis indicated that increased DAP3 expression was linked to poorer prognosis. Cox regression analysis identified DAP3 expression as a prognostic indicator for overall survival in HCC patients. *In vitro*, we performed functional experiments to examine the role of DAP3 in HCC progression. The upregulation of DAP3 expression was found to suppress the proliferation, migration, and invasion of HCC cells while promoting apoptosis. These findings underscored the strong association between elevated DAP3 expression and adverse outcomes in HCC patients, highlighting the potential significance of DAP3 in driving HCC progression.

Cancer is a multifaceted disease characterized by intricate reciprocal interactions between tumor cells and the immune system (30). The tumor microenvironment (TME) mainly consists of distinct immune cell populations in tumor islets and highly influences tumor growth, metastatic spread, and response to treatment (31, 32). Due to the poor prognosis following standard treatment, immunotherapy has been extensively investigated as an alternative treatment option (33). Cancer immunotherapies, particularly immune checkpoint blockade therapy, have fundamentally transformed cancer treatment by facilitating complete and sustained responses and have now emerged as a standard approach for a variety of malignancies (34). Regrettably, only a limited number of patients with specific cancer types respond to immunotherapy, possibly due to inadequate immune activation needed to detect tumor-specific antigens (35). Therefore, it is essential to identify additional potential therapeutic targets. Our initial exploration demonstrated that high DAP3 expression was closely associated with immune cell infiltration, immune evasion mechanisms, and gene expression patterns, especially impacting immunotherapy response and immune evasion, indicating that DAP3 plays a regulatory role in the tumor microenvironment and may serve as an important biomarker for immunotherapy in the clinic.

Genetic mutations can disrupt normal cellular functions, including proliferation, differentiation, and apoptosis, ultimately leading to tumor formation (36). Different types of cancer are often associated with specific genetic mutations, which can influence cell behavior, treatment responsiveness, and immune recognition (37). In our study, we observed that high DAP3 expression was linked to increased genomic mutation rates, homologous recombination defects, and copy number variations. These findings revealed a potential role for DAP3 in driving genomic alterations within tumors.

Currently, high mortality and recurrence rates continue to pose significant challenges to the advancement of effective treatment options for this disease (38, 39). The prognosis for HCC patients remains poor, with a 5-year overall survival rate of only 12% (40). There is an urgent need for novel prognostic predictors and the development of more robust prognostic models for HCC. We ultimately constructed a new prognostic model for HCC (41), and demonstrated that both the risk score and the nomogram were effective predictive tools that can accurately predict patient survival when combined with clinical characteristics. Moreover, to overcome drug resistance and improve the clinical outcomes of HCC patients, we identified potential chemotherapy drugs and found that these drugs had lower AUC values in the high-risk score group than in the low-risk score group and were negatively correlated with the risk score, indicating that chemotherapy drugs were more sensitive in the high-risk score group. Nevertheless, there are some limitations to the drug sensitivity findings; although the 20 candidate drugs exhibited increased drug sensitivity in patients with high-risk scores, the above analysis alone does not support the conclusion that these drugs have therapeutic effects on HCC.

In summary, DAP3 plays a regulatory role in both the tumor microenvironment and genomic alterations within tumors. Our findings confirmed that the risk score and nomogram model were effective predictive tools for HCC when combined with clinical characteristics. *In vitro* studies demonstrated that DAP3 could regulate proliferation, apoptosis, and metabolism in HCC cells. However, this study had several limitations. Although DAP3 shows promising potential as a biomarker and therapeutic target in liver cancer, the specific pathway for its clinical translation remains unclear. A larger sample size and more experimental validation would strengthen the conclusions and help establish more robust evidence for the role of DAP3 in liver cancer.

## Conclusions

This study highlights DAP3 as a significant factor in cancer prognosis and immune regulation, offering insights that could inform future therapeutic strategies.

## Data Availability

The raw data supporting the conclusions of this article will be made available by the authors, without undue reservation.

## Supplementary material

Supplementary Figure 1 Correlation between DAP3 and immune-related functions in HCC: **(A)** The impact of DAP3 expression levels on Sorafenib treatment response. **(B)** ROC analysis of DAP3 expression and Sorafenib treatment response.

Supplementary Figure 2 A single-cell expression atlas and the identification of DAP3 in HCC by scRNA-seq: **(A)** Boxplot comparing CytoTRACE2 scores in the DAP3+ and DAP3− groups. **(B)** Correlation analysis between DAP3 expression levels and CytoTRACE2 scores. **(C)** UMAP plot illustrating the heterogeneity of CytoTRACE2 scores among different cell populations. **(D)** Volcano plot displaying differentially expressed genes between DAP3+ and DAP3− tumor cells. **(E)** Bubble plot showing the top enriched pathways. **(F)** Heatmap of metabolic pathway activity in DAP3+ and DAP3− groups across primary and relapsed tumor samples.

Supplementary Figure 3 Time-dependent ROC curve analysis to assess the predictive efficacy of the prognosis signature based on datasets.
