# Supplementary figures and images for: Identification of DAP3 as candidate prognosis marker and potential therapeutic target for hepatocellular carcinoma
Source: Front Immunol. 2025 Feb 20;16:1528853. doi: 10.3389/fimmu.2025.1528853 (PMC11882876; doi:10.3389/fimmu.2025.1528853)

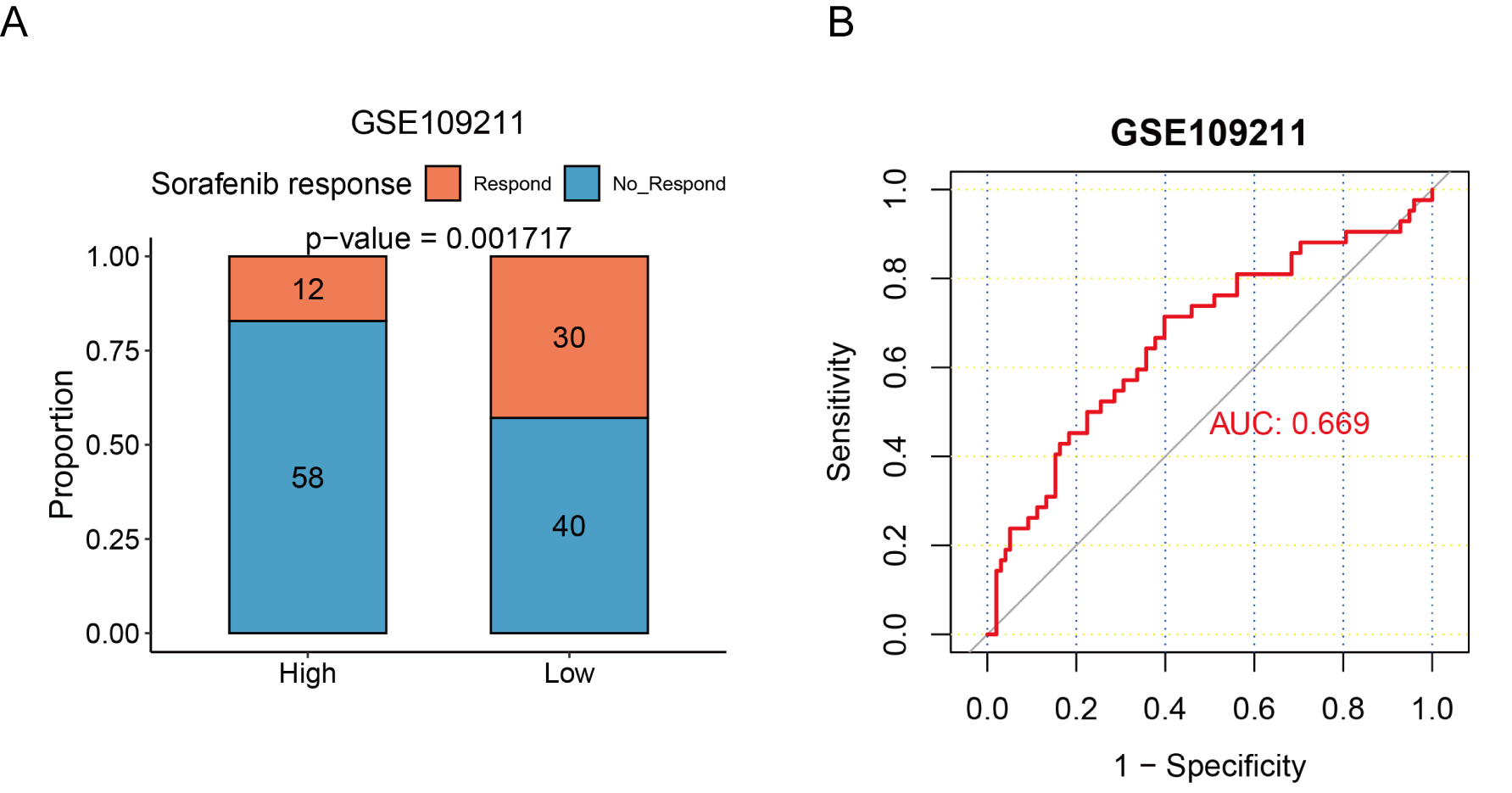

Supplement: Supplementary Figure 1 — Correlation between DAP3 and immune-related functions in HCC: (A) The impact of DAP3 expression levels on Sorafenib treatment response. (B) ROC analysis of DAP3 expression and Sorafenib treatment response. [file Image1.tif]

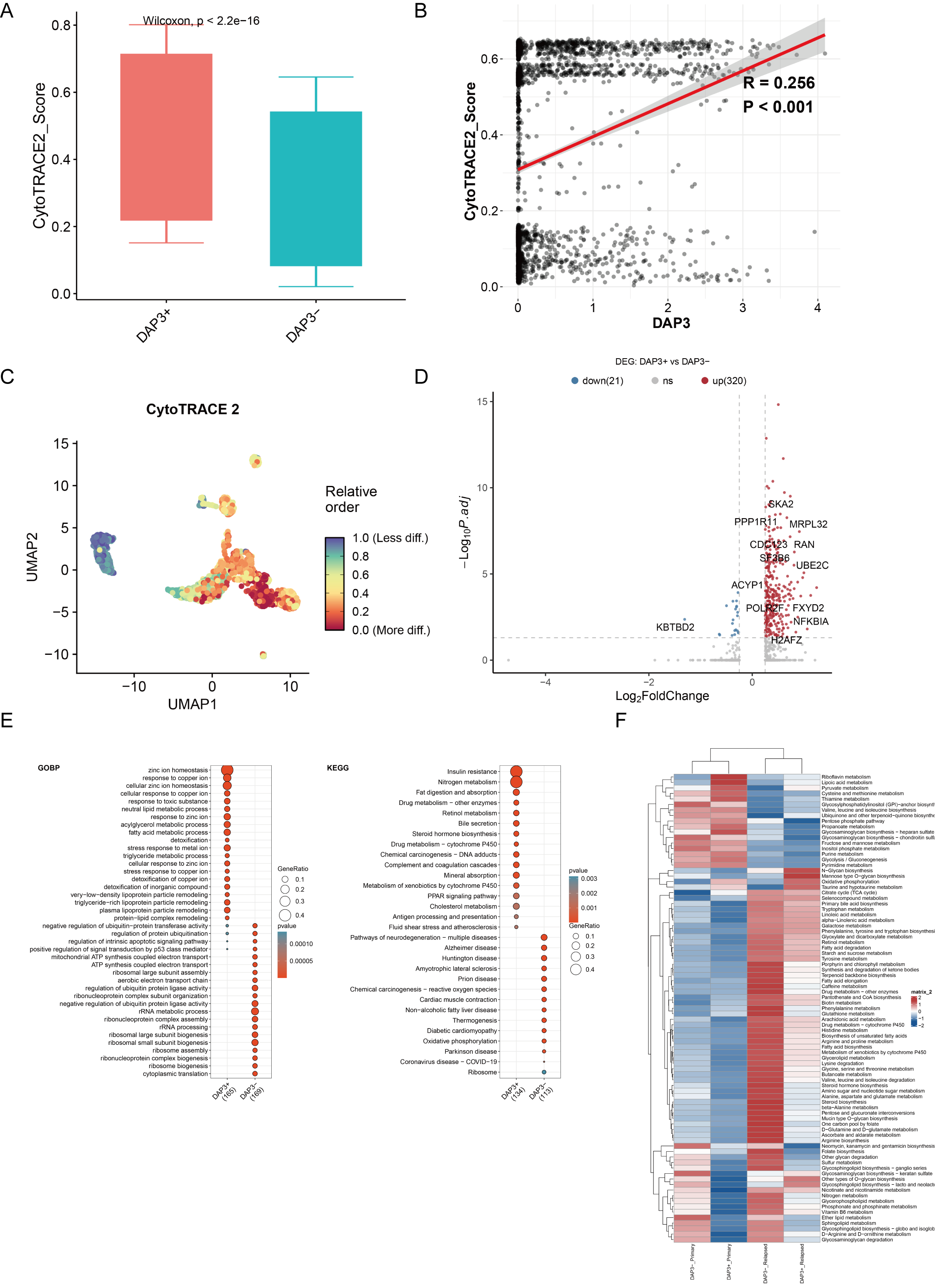

Supplement: Supplementary Figure 2 — A single-cell expression atlas and the identification of DAP3 in HCC by scRNA-seq: (A) Boxplot comparing CytoTRACE2 scores in the DAP3+ and DAP3− groups. (B) Correlation analysis between DAP3 expression levels and CytoTRACE2 scores. (C) UMAP plot illustrating the heterogeneity of CytoTRACE2 scores among different cell populations. (D) Volcano plot displaying differentially expressed genes between DAP3+ and DAP3− tumor cells. (E) Bubble plot showing the top enriched pathways. (F) Heatmap of metabolic pathway activity in DAP3+ and DAP3− groups across primary and relapsed tumor samples. [file Image2.tif]

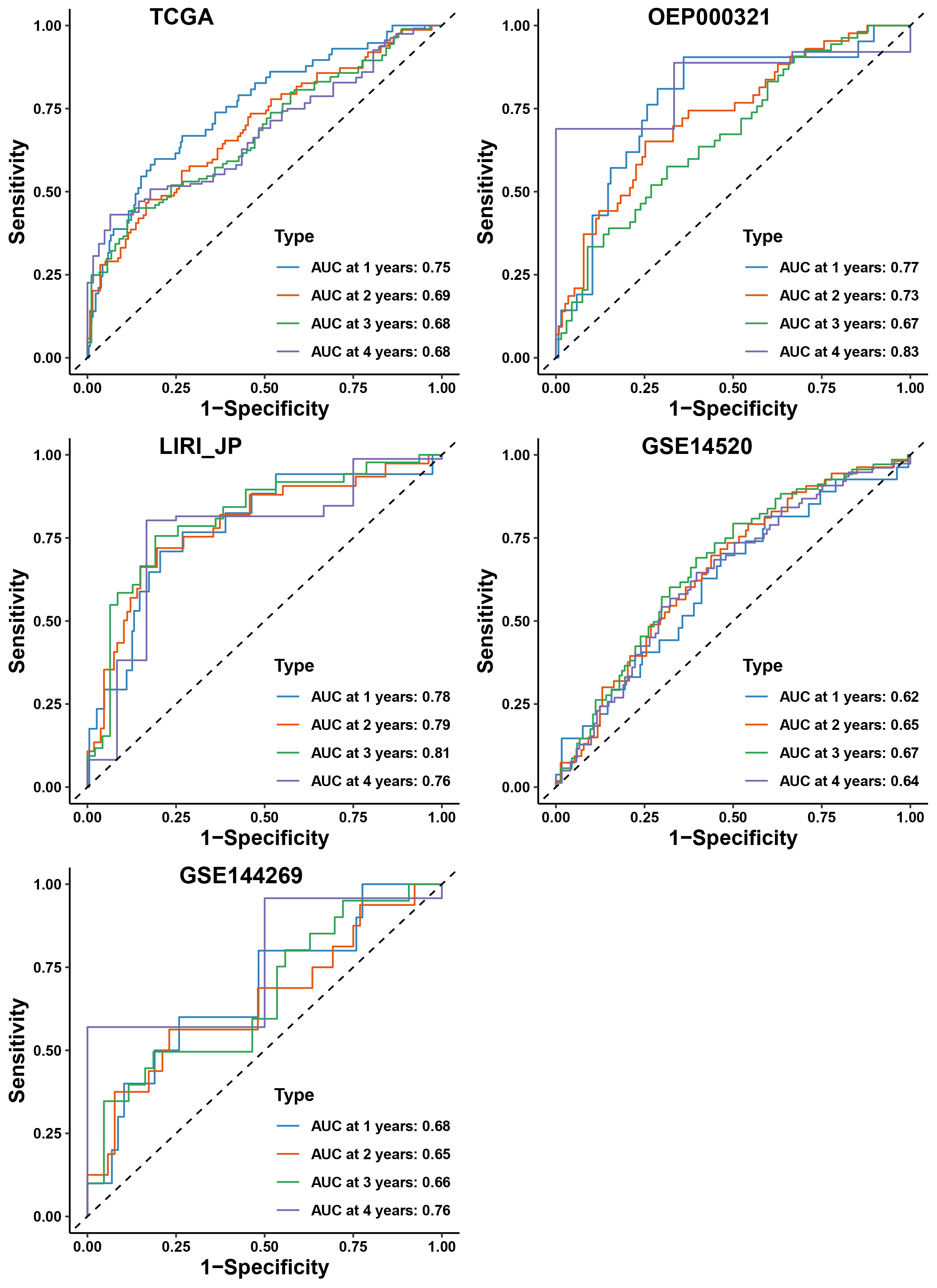

Supplement: Supplementary Figure 3 — Time-dependent ROC curve analysis to assess the predictive efficacy of the prognosis signature based on datasets. [file Image3.tif]
